# Dynamic Resource Allocation and Access Class Barring Scheme for Delay-Sensitive Devices in Machine to Machine (M2M) Communications

**DOI:** 10.3390/s17061407

**Published:** 2017-06-15

**Authors:** Ning Li, Chao Cao, Cong Wang

**Affiliations:** College of Communications Engineering, PLA University of Science and Technology, Nanjing 210007, China; lining_friend@sina.com

**Keywords:** M2M communication, delay-sensitive devices, ACB mechanism, resource allocation, Markov chain

## Abstract

Supporting simultaneous access of machine-type devices is a critical challenge in machine-to-machine (M2M) communications. In this paper, we propose an optimal scheme to dynamically adjust the Access Class Barring (ACB) factor and the number of random access channel (RACH) resources for clustered machine-to-machine (M2M) communications, in which Delay-Sensitive (DS) devices coexist with Delay-Tolerant (DT) ones. In M2M communications, since delay-sensitive devices share random access resources with delay-tolerant devices, reducing the resources consumed by delay-sensitive devices means that there will be more resources available to delay-tolerant ones. Our goal is to optimize the random access scheme, which can not only satisfy the requirements of delay-sensitive devices, but also take the communication quality of delay-tolerant ones into consideration. We discuss this problem from the perspective of delay-sensitive services by adjusting the resource allocation and ACB scheme for these devices dynamically. Simulation results show that our proposed scheme realizes good performance in satisfying the delay-sensitive services as well as increasing the utilization rate of the random access resources allocated to them.

## 1. Introduction

With the rapid development of the Internet of Things (IoT) [1], one of the major drivers of cellular networks is machine-to-machine (M2M) communication [2,3]. M2M communications, also known as machine-type communications (MTC), means the communications of machine devices without human intervention [4]. Many devices may be triggered almost simultaneously and attempt to access the base station through the Random Access Channel (RACH). M2M communications, as a critical part of the development of the IoT, are crucial to efficient data transmission from machine devices to networks for various IoT applications such as smart metering, health-care, smart home appliances, surveillance, security and logistics tracking [5,6,7]. 

M2M services have great market potential because of their wide range of applications. Recent market reports forecast that in 2020, 50 billion of machine devices are expected to be deployed and connected to the network to serve the IoT [8]. It should be noted that compared to traditional human-to-human (H2H) communications, M2M communications have many different characteristics. A typical feature of M2M services is that they consume little bandwidth with subtle impact on Radio Access Network (RANs) [9]. Nevertheless, such kinds of communications generally involve an extremely large number of MTC devices to support various applications. Therefore, a critical challenge of MTC is how to tackle the network degradation caused by small data transmissions and vast heterogeneous applications.

The traditional cellular network, which is originally engineered for H2H communications, has been considered unsuitable to handle the unique characteristics of M2M applications [10,11]. It needs to be specifically adapted to match the Quality of Service (QoS) requirements of M2M applications. In order to facilitate M2M communications through existing cellular networks such as Long Term Evolution-Advanced (LTE-A) [12], the Third Generation Partnership Project (3GPP) organization has initiated related study items and working groups. Considering the characteristics of high density, intermittent transmission and battery powered for M2M communications, one of the major problem of M2M communications is congestion vulnerability. When a massive number of MTC devices attempt to access the eNodeB simultaneously, it will inevitably cause severe network congestion due to the limited number of Physical Random Access Channel (PRACH) resources in a single cell system [13]. The network congestion and overloading will inevitably increase delays, cause packet loss and even lead to the service interruption of H2H communications [10].

Due to diverse application scenarios, QoS requirements in M2M communications exhibit a relatively wider range [14]. Therefore, another valuable research issue is how to meet the growing diversity of QoS requirement for MTC devices. When we measure QoS requirements for various M2M sevices, delay requirement is a major concern. Some M2M applications, such as smart grid and fire alarm, have very stringent delay requirements [15]. It is likely to cause incalculable loss of property and a threat to life security if we cannot effectively meet the QoS requirements of these services. Providing effective access policies for such devices is one of the research focuses.

The remainder of this paper is organized as follows: in Section 2, we explain preliminaries on the related works and the random access procedure in LTE-A system. In Section 3, we introduce the clustered structure and the conceptual design of our proposed scheme in detail. In Section 4, we introduce the analytical model and the derivation of performance parameters. In Section 5, the performance of our scheme is evaluated by comparing our proposed scheme with typical traditional schemes. The paper is concluded in Section 6.

## 2. Preliminaries

### 2.1. Related Works

According to key problems in recent M2M communications, there are many studies being carried out to alleviate RAN overload and network congestion [11]. These studies include applying the slotted aloha scheme, the pull-based scheme, the MTC device back-off scheme, and the access class barring (ACB) scheme, among which ACB scheme is currently regarded as the major solution in M2M communications. The key of ACB scheme is to let the eNodeB broadcast a parameter to all MTC devices. When an MTC device tries to initiate a transmission, it generates a random number between 0 and 1, and compares the generated number with the ACB factor broadcast by eNodeB. If the number is less than the ACB factor, the MTC device proceeds to access the eNodeB. Otherwise, it needs to backoff temporarily.

For existing research on ACB scheme, most of the studies emphasize the estimation of random access load and the dynamic adjustment of ACB parameters. Reference [16] utilized a PID controller to adaptively adjust the ACB factor. A Markov-Chain-based traffic-load estimation scheme according to the network collision status is developed by [17]. Reference [18] presented two dynamic access class barring (D-ACB) algorithms for fixed and dynamic preamble allocation schemes to determine the ACB factors, providing an effective method of reducing total service time. 

Except for ACB scheme, rational allocation of RACH resources is also an effective solution to tackle the RAN overload [19,20,21]. In [19], the authors proposed a dynamic RACH preamble allocation scheme based on the value of the ACB factor. A two stage resource allocation scheme is presented by [20]. By setting two layer ACB scheme, the proposed scheme can remarkably improve resource efficiency. Reference [21] studied the scene where M2M devices coexist with H2H devices, M2M User Equipments (UEs) form coalitions and perform relay transmission with an objective to reduce network congestion.

As different M2M devices have various QoS requirements, a Multiple Access Class Barring (MACB) mechanism is presented by [22]. The main idea of MACB mechanism is to set distinguishing access priorities for different services. Reference [23] proposed an Extended Access Barring (EAB) mechanism to enhance the performance of ACB scheme. The basic idea of both MACB and EAB schemes is that the delay-tolerant devices are not permitted to access the network while the delay-sensitive ones are enable to request access attempts as long as MACB is activated in case of network congestion. However, an evident drawback of MACB is that it does not realize the partition of RACH resources. Moreover, it is worth noting that in real systems, the number of delay-sensitive devices is less than delay-tolerant ones. It ignores the communication quality of delay-tolerant devices. 

Thus, we need an intelligent solution for efficient resource management between coexisting delay-sensitive and delay-tolerant services to address the aforementioned problems. In this paper we propose an optimal scheme that combines an ACB scheme and RACH resource separation for two given clusters which are divided according to the delay requirement of different devices. The originality of our work is that our scheme adjusts the ACB factor and the number of preambles allocated to two clusters dynamically from the perspective of delay-sensitive services. Simulation results show that our proposed scheme shows good performance in meeting the delay requirements as well as increasing the utilization rate of the RACH resources allocated to them. High utilization rate ensures that more resources are left for delay-tolerant devices.

### 2.2. Random Access Procedure in LTE-A System

In the Long Term Evolution-Advanced (LTE-A) system, a Random Access (RA) method, called Random Access Procedure, has been proposed for MTC [24]. The Random Access Procedure is identified as a key step for initial access [25]. In the Random Access Procedure, two uplink channels are required, i.e., Physical Random Access Channel (PRACH) and Physical uplink Shared Channel (PUSCH). PRACH is used for preamble transmission, and user data is scheduled to be transmitted through PUSCH. Random Access Channels (RACHs) are time-frequency resource blocks (RBs) repeated in the system periodically. There is a set of codes called preambles which are shared by all users in their random access. Each node requesting an uplink channel transmits a random access preamble in a RACH. There are two types of access modes in RACHs [3]. The first one is contention-free, eNodeB allocates a dedicate preamble sequence to each UE to ensure that no other UE will use the same preamble in the same PRACH at the same time. In this case, there is no collision in random access procedure [26]. The second type is contention-based, where a user selects a preamble randomly from the set of available preambles. In this case, two nodes may select the same preamble, resulting in a conflict. Most of the current studies are discussed in contention-based random access.

The traditional contention-based access mode is comprised of four steps. The corresponding signaling is Msg1–Msg4:

- Msg1: preamble transmission. Once an MTC device launches an access request to the RACH, it randomly selects a preamble with equal probability and transmits the selected preamble to the eNodeB via PRACH (i.e., the same uplink time-frequency resources). When two or more nodes select identical preambles and send them at the same time, there could be a collision.
- Msg2: random access response. If a preamble has been received correctly, the eNodeB computes an identifier and then transmits a random access response (RAR) to the UE devices. The RAR includes a RA preamble identifier (ID), an uplink grant for MSG3, timing alignment (TA) command for corresponding UEs, and assignment of a temporary identifier (the cell radio network temporary identifier, CRNTI). UE is expected to receive RAR within a timing window.
- Msg3: data transmission. A UE first finds its random access response by looking up the index of the preamble it has used in its random access request, and then uses the dedicated resource block (RB) on PUSCH to transmit a Connection Request message with a UE identifier to the eNodeB. If two or more UEs select an identical preamble in Step 1, they will implement uplink scheduling in the same RBs, thus scheduled message will not be correctly decoded by eNB due to the co-channel interference. This section is the main reason of random access conflict.
- Msg4: contention resolution. Upon reception of a Connection Request in Step 3, the eNodeB transmits a Connection Resolution message as an response to Step 3. Therefore, if a device does not receive Step 4, it will indicate a failure in the Contention Completion and launch a new access request after a random backoff.

**Figure 1:**
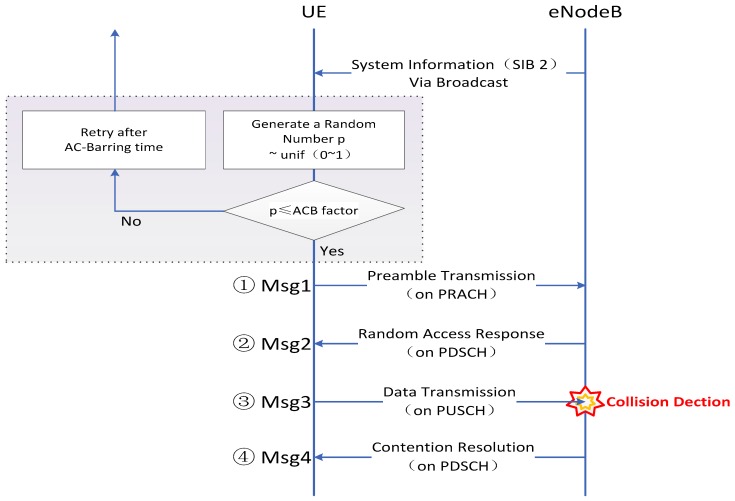
Random access procedure through ACB mechanism.

As we have introduced the ACB scheme in Section 2.1, the Random Access Procedure through the ACB mechanism is depicted in Figure 1.

## 3. Dynamic Resource Allocation and ACB Scheme for Delay-Sensitive Devices

**Figure 2:**
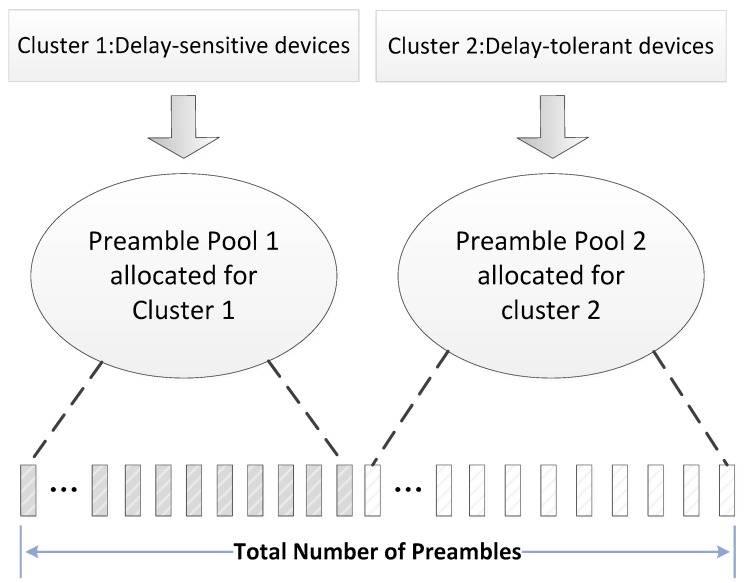
The diagram of conceptual design.

In this section, we address the implementations of our scheme, as depicted in Figure 2. Specifically, our scheme is composed of two parts. Firstly, on the basis of delay requirements, we consider that MTC devices are classified into two clusters. Secondly, the most critical design is achieved by dynamically adjusting the value of ACB factor and the number of preambles allocated to the two clusters.

### 3.1. Clustured Structure

Delay-sensitive devices utilize the preamble resources occasionally due to the lower incidence of such services, which results in smaller traffic loads compared with delay-tolerant ones. Considering this, we classify those devices that attempting to access the network into two clusters according to their delay requirements. As depicted in Figure 2, we divide the available preambles which are reserved for contention-based random access procedure into two pools (marked as Pool 1 and Pool 2). The preambles in Pool 1 are dedicated for DS devices while preambles in Pool 2 serve the delay-tolerant ones. The number of preambles in each pool is not fixed and it can be adjusted by eNodeB dynamically with the change of network overload. It is worth noting that the total amount of the preambles in the two pools remains unchanged. Considering the distinct communication requirements of DS and DT devices, we set different ACB factors for the devices in different clusters. As general delay-tolerant devices can tolerate high latency, we give priority to DS devices and focus on ACB scheme and resource allocation from the perspective of this type of devices. In addition, in order to avoid all RA resources are occupied by DS devices, we set an upper bound which is denoted as $L_{avail\_DS}$ for the number of preambles in Pool 1. Furthermore, we define $L_{total}$ as the total number of available preambles for DS and DT devices.

### 3.2. Proposed Optimization Scheme

In this paper, we assume that random access requests are all initialed at the beginning of a slot. In order to express our proposed scheme clearly, we present the concept of active device. Here, an active device is defined as a DS device which has a packet to send to eNodeB at the beginning of an RA slot. The active devices consist of two parts, i.e., devices which newly arrived in the current slot and devices which are barred and collided in the previous slot. In addition, due to the function of ACB mechanism, only part of the active devices can transmit preambles, we define this part of devices as contending devices.

Let N denote the number of active devices that arrived in an access slot, $N_{pa}$ denote the expected number of DS devices that pass through the ACB mechanism. Reviewing the implementation process of ACB scheme which has been introduced in Section 2.1, we obtain the expression of $N_{pa}$ as:

(1) $$N_{pa}=N\cdot p_{ACB}$$

where $p_{ACB}$ denotes the ACB factor for DS devices.

Let $S_{l}=0$, $S_{l}=1$ and $S_{l}=c$ respectively denote the cases that a random selected preamble l is idle (i.e., is selected by none of the users), is successfully transmitted (i.e., is selected by exactly one user) and is in conflict (i.e., is selected by more than one user). The probability that only one user among $N_{pa}$ contending devices selects preamble l is:

(2) $$P\left(s_{l}=1\right)=\left(\begin{array}{c} N_{pa}\\ 1 \end{array}\right)\frac{1}{L_{DS}}{\left(1-\frac{1}{L_{DS}}\right)}^{N_{pa}-1}$$

here $L_{DS}$ indicates the number of preambles allocated to DS devices, i.e., the number of preambles in Pool 1.

It should be noted that preamble utilization rate $P_{L\_\mathrm{succ}}$, which represents the ratio of the number of successfully transmitted preambles for DS devices to the number of total preambles allocated to DS devices, is the same as the probability that a preamble is selected by exactly one user as shown in Equation (2). Thus, we can derive the expression of $P_{L\_\mathrm{succ}}$ as:

(3) $$P_{L\_\mathrm{succ}}=P\left(s_{l}=1\right)=\left(\begin{array}{c} N_{pa}\\ 1 \end{array}\right)\frac{1}{L_{DS}}{\left(1-\frac{1}{L_{DS}}\right)}^{N_{pa}-1}$$

Through derivation we can judge that $P_{L\_\mathrm{succ}}$ is a ∩ shape of $L_{DS}$ for a fixed $N_{pa}$, $P_{L\_\mathrm{succ}}$ can get its maximum value when $L_{DS}=N_{pa}$ [27]. 

We define $P_{D\_succ}$ as the probability of a DS device successfully accessing the network, it can be derived as follows:

(4) $$P_{D\_succ}=p_{ACB}\cdot P_{access}$$

where $P_{access}$ denotes the probability that a contending DS device which passes through the ACB mechanism can successfully access the network, i.e., the preamble chosen by the device is not selected by any other UEs. We can obtain $P_{access}$ as:

(5) $$P_{access}=\frac{\left(\begin{array}{c} N_{pa}\\ 1 \end{array}\right)\cdot {\left(1-\frac{1}{L_{DS}}\right)}^{N_{pa}-1}}{N_{pa}}={\left(1-\frac{1}{L_{DS}}\right)}^{N_{pa}-1}$$

We define $T_{delay}$ as the access delay, i.e., the delay between the first access attempt and the completion of a successfully preamble transmission for a DS device. In legacy RA process, each device blocked by ACB mechanism and preamble collision will reattempt access after a random backoff. In our paper, we assume that all the devices blocked in a certain RA slot will launch a new access request in the next coming RA slot and there is no retry limit for random access, thus the average value of access delay $T_{delay}$ can be derived as:

(6) $$T_{delay}={\sum_{r=1}^{\infty }r\cdot P_{D\_succ}\cdot \left(1-P_{D\_succ}\right)}^{r-1}\cdot T_{slot}=\frac{T_{slot}}{P_{D\_succ}}$$

where $T_{slot}$ denotes the length of a random access slot, r denotes the number of access attempts initiated by the DS device before the preamble selected by the device is successfully transmitted. 

Substituting Equations (4)–(6), access delay can be determined as:

(7) $$T_{delay}=\frac{T_{slot}}{p_{ACB}\cdot {\left(1-\frac{1}{L_{DS}}\right)}^{N_{pa}-1}}$$

Now, let us investigate the optimization strategy for DS devices. Considering the characteristics of DS services, we mainly think about two optimization parameters:

1. Average access delay of delay-sensitive devices.
2. Preamble utilization rate for delay-sensitive devices.

The significance for discussing the above two parameters are as follows: firstly, by studying the average access delay, we can effectively meet the basic QoS requirements of DS devices; secondly, by studying the preamble utilization rate, we can achieve the most efficient use of PRACH resources for DS devices, thus leaving more preambles for Pool 2. 

There have been a lot of articles about estimating the network load [17,18,28,29,30,31], the number of attempting devices can be estimated using the number of idle preambles or the number of collision preambles. Therefore, we do not discuss how to estimate network load in our paper. In the following discussion, we assume that the eNodeB knows the actual number of DS and DT devices that attempt to access the network. Our focus is on the joint optimization of the ACB mechanism and the preamble allocation scheme for DS devices. 

It has been mentioned in the first part of the paper that $P_{L\_\mathrm{succ}}$ is maximized if $L_{DS}$ is equal to $N_{pa}$. However, we must consider the characteristics of the LTE-A system, i.e., the maximum number of available preambles for DS devices. In order to maintain high preamble utilization rate of DS devices, the number of contending devices which can be controlled by ACB mechanism needs to be minimized, as the number of contending devices increases, more preambles are required to maximize the preamble utilization rate. In addition, a reasonable setting of the value of $L_{DS}$ and $p_{ACB}$ is needed to increase the delay performance. We have defined $L_{avail\_DS}$ as the maximal number of available preambles for DS devices in Section 3.1. According to the number of active devices (N) in the slot, the investigation of our proposal can be divided into two cases: one is $N\leq L_{avail\_DS}$ and the other is $N>L_{avail\_DS}$. By theoretical analysis, we can obtain the optimal values of N and $p_{ACB}$ for each case. We discuss these two situations separately:

1. $N\leq L_{avail\_DS}$

In this situation, ACB mechanism is not necessary as we have sufficient preamble resources for DS devices. Therefore, our principle is to deal with as many DS devices as possible for each slot. For the purpose of enabling more DS devices to transmit preambles, we set $p_{ACB}$ to the maximum value $p_{ACB}^{*}=1$, where $p_{ACB}^{*}$ denotes the optimal value of $p_{ACB}$. Our problem can be formulated as:

(8) $$L_{DS}^{*}=\arg\underset{0\leq L_{DS}\leq L_{avail\_DS}}{\max}P_{L\_succ}\;s.t.:T_{delay}\leq D_{req}$$

where $L_{DS}^{*}$ is defined as the optimal value of $L_{DS}$. $D_{req}$ is defined as the delay requirement of DS devices. Considering that $N_{pa}=N$ when $p_{ACB}$ is set to 1, by substituting $N_{pa}=N$, $p_{ACB}=1$ into Equation (7), the set of $L_{DS}$ to meet the delay requirement can be determined as:

(9) $$L_{set}=\left\{L_{DS}|\frac{T_{slot}}{{\left(1-\frac{1}{L_{DS}}\right)}^{N-1}}\leq D_{req},0<L_{DS}\leq L_{avail\_DS}\right\}$$

reviewing Equation (8), we can subsequently obtain the optimal value of $L_{DS}$ as:

(10) $$L_{DS}^{*}=\arg\underset{L_{DS}\in L_{set}}{\max}P_{L\_succ}$$

For the special case that $L_{set}$ calculated from Equation (9) is ∅, we take $L_{DS}^{*}=N$ referring to the conclusion we have mentioned before that $P_{L\_\mathrm{succ}}$ can achieve its maximal value when $L_{DS}=N_{pa}$.

2. $N>L_{avail\_DS}$

In this situation, the amount of preamble resources is not sufficient for DS devices that attempt to access the network, thus we take $L_{DS}^{*}$ as the maximum value $L_{avail\_DS}$ to serve more devices. Our principle is to make use of the ACB mechanism for the reason that the number of contending devices can be controlled appropriately by adjusting the ACB factor. Thus, the problem can be formulated as:

(11) $$p_{ACB}^{*}=\arg\underset{0\leq p_{ACB}\leq 1}{\max}P_{L\_succ}\;s.t.:T_{delay}\leq D_{req},$$

where $p_{ACB}^{*}$ is the optimal value of $p_{ACB}$.

We know the average number of DS devices that pass the ACB mechanism is $N_{pa}=N\cdot p_{ACB}$. Therefore, reviewing the expression of access delay in Equation (7), we are able to obtain the set of $p_{ACB}$ to meet the delay requirement as follows:

(12) $$p_{set}=\left\{p_{ACB}|\frac{T_{slot}}{p_{ACB}\cdot {\left(1-\frac{1}{L_{avail\_DS}}\right)}^{N\cdot p_{ACB}-1}}\leq D_{req},0<p_{ACB}\leq 1\right\}$$

by substituting Equation (12) into Equation (11), the optimal value of $p_{ACB}$ can be written as:

(13) $$p_{{}_{ACB}}^{*}=\arg\underset{p_{ACB}\in p_{set}}{\max}P_{L\_succ}$$

Similar to the first situation, for the special case when $p_{set}$ obtained by Equation (12) is ∅, we take $p_{ACB}^{*}=\frac{L_{avail\_DS}}{N}$ to maximum the preamble utilization rate. 

In order to facilitate the understanding of the proposed control scheme which has been discussed in the previous paragraph, our algorithm to obtain $L_{DS}^{*}$ and $p_{ACB}^{*}$ when the number of attempting DS devices in a certain RA slot is N is summarized in Algorithm 1.

**Algorithm 1** Proposed dynamic resource allocation and ACB scheme in a slot1: N: number of DS devices attempt to access2: $p_{ACB}$: ACB factor3: $L_{DS}^{*}$: optimal number of preambles allocated to DS devices4: $p_{ACB}^{*}$: optimal value of ACB factor for DS devices5: $L_{avail}$: number of available preambles6: **if**
$N\leq L_{avail\_DS}$
**then**7:   Set $p_{ACB}^{*}=1$;8:   Compute $L_{set}$ through Equation (9);9:     **if**
$L_{set}=\emptyset$
**then**10:     $L_{DS}^{*}=N$;11:     **else**12:     Compute $L_{DS}^{*}$ through Equation (10);13:     **end if**14: **else if**
$N>L_{avail\_DS}$
**then**15:   Set $L_{DS}^{*}=L_{avail\_DS}$16:   Compute $p_{set}$ through Equation (12);17:     **if**
$p_{set}=\emptyset$
**then**18:      pACB*=Lavail_DSN;19:     **else**20:      Compute $p_{ACB}^{*}$ through Equation (13);21:     **end if**22: **end if**

## 4. Analysis Model

**Figure 3:**
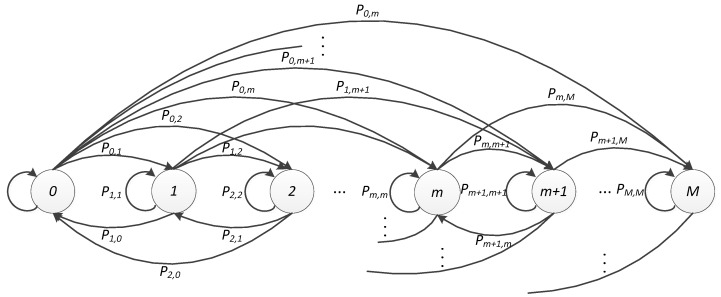
Markov chain for the number of access requests at the beginning of a slot.

In this section, we derive an analytical model for evaluating the performance of the proposed scheme. We use a Markov chain to analyze each state of the random access slot. The state transition diagram is depicted in Figure 3. The state of the Markov chain represents the number of DS devices right before the start of an RA slot, i.e., the number of active devices in a slot. In this model, state M means that there are greater than or equal to M active devices arrive at the slot. Since we are considering the simultaneous DS devices’ access environment, the setting value of M is far greater than $L_{avail\_DS}$ under the background of LTE-A system.

In order to obtain average access delay, average preamble utilization rate and average number of active devices in each slot, etc. We need to calculate the probability of each state by steady-state equations. Therefore, the first thing we need to do is calculating the steady-transition matrix P. Let $P_{m,n}$ denote the transition probability that the state transferred from m to n. We can derive:

(14) $$P_{0,n}=A_{n},\;\mathrm{for}\;n\in \left(0,M-1\right)$$

(15) $$P_{0,M}=A_{\geq M}$$

where $A_{n}$ denotes the probability that there are n devices arrived at the access slot, $A_{\geq M}$ as denotes the probability that more than M devices arrive at the slot. 

We define $B_{m,s}$ and $F_{m,s,t}$ as the probabilities that s devices are blocked by ACB mechanism among m devices and t devices are collided in the preamble transmission section among m−s contending devices. Then the state transition probability $P_{m,n}$ can be represented as follows:

(16) $$P_{m,n}=\sum_{s=0}^{m}B_{m,s}\cdot \left(\sum_{t=0}^{m-s}F_{m,s,t}\cdot A_{n-s-t}\right),\;\mathrm{for}\;m\in \left(1,M-1\right),\;n\in \left(m,M-1\right)$$

(17) $$P_{m,n}=\sum_{s=0}^{m}B_{m,s}\cdot \left(\sum_{t=0}^{m-s}F_{m,s,t}\cdot A_{n-s-t}\right),\;\mathrm{for}\;m\in \left(1,M\right),\;n\in \left(m-\min\left(m,L_{avail}\right),m-1\right)$$

(18) $$P_{m,M}=\sum_{s=0}^{m}B_{m,s}\cdot \left(\sum_{t=0}^{m-s}F_{m,s,t}\cdot A_{\geq n-s-t}\right),\;\mathrm{for}\;m\in \left(1,M\right)$$

In our paper, we use Poisson distribution as our arrival model, which is widely used to analyze a slotted ALOHA [32]. The arrival rate of DS devices in our paper is λ, thus we can obtain:

(19) $$A_{n}=\frac{{\left(\lambda T\right)}^{n}}{n!}e^{-\lambda T},n=0,1,2,...$$

(20) $$A_{\geq M}=1-\sum_{n=0}^{M-1}A_{n}$$

Let $p_{ACB,m}^{*}$ denote the optimal ACB factor derived from Algorithm 1 when the number of active devices is m, then $B_{m,s}$ can be written as:

(21) $$B_{m,s}=\left(\begin{array}{c} m\\ s \end{array}\right){\left(1-p_{ACB,m}^{*}\right)}^{s}{\left(p_{ACB,m}^{*}\right)}^{m-s}$$

In addition, we can obtain the expression of $F_{m,s,t}$ as:

(22) $$F_{m,s,t}=\left(\begin{array}{c} m-s\\ t \end{array}\right){\left(p_{fail,m,s}\right)}^{t}{\left(1-p_{fail,m,s}\right)}^{m-s-t}$$

where $p_{fail,m,s}$ denotes the probability that a DS device fails to access the network because of contention of PRACH resources with other (m−s−1) devices. Knowing the number of allocated preambles, we can further obtain the expression of $p_{fail,m,s}$:

(23) $$p_{fail,m,s}=\sum_{r=1}^{m-s-1}\left(\begin{array}{c} m-s-1\\ r \end{array}\right){\left(L_{DS,m}^{*}\right)}^{r}{\left(1-L_{DS,m}^{*}\right)}^{m-s-1-r}$$

here $L_{DS,m}^{*}$ denotes the calculated optimal number of $L_{DS}$ obtained by Algorithm 1 when the number of active devices is m.

By substituting Equations (19)–(23) into Equations (14)–(18), we can get the state transition matrix:

(24) $$P=\left[\begin{array}{cccccc} P_{0,0} & P_{0,1} & \cdots & P_{0,m} & \cdots & P_{0,M}\\ P_{1,0} & P_{1,1} & \cdots & P_{1,m} & \cdots & P_{1,M}\\ \vdots & \vdots & \ddots & \vdots & \vdots \\ P_{m,0} & P_{m,1} & \cdots & P_{m,m} & \cdots & P_{m,M}\\ \vdots & \vdots & \vdots & \ddots & \vdots \\ P_{M,0} & P_{M,1} & \cdots & P_{M,m} & \cdots & P_{M,M} \end{array}\right]$$

The steady-state equation is listed as follows:

(25) $$\{\begin{array}{c} \vec{\pi }\cdot P=\vec{\pi }\\ \sum_{i=1}^{M}\pi_{i}=1 \end{array}$$

where $\vec{\pi }$ denotes the steady-state probability vector, i.e., $\vec{\pi }=\left\{\pi_{0},\pi_{1},\pi_{2},\pi_{3},\cdots ,\pi_{M}\right\}$. Solving the Equation (25), we can obtain $\vec{\pi }$.

Combining Equation (1) with Equation (3), the preamble utilization rate in state m can be written as:

(26) $$P_{L\_succ,m}=\frac{N_{pa}{\left(1-\frac{1}{L_{DS,m}^{*}}\right)}^{N_{pa}-1}}{L_{DS,m}^{*}}=\frac{m\cdot p_{ACB,m}^{*}{\left(1-\frac{1}{L_{DS,m}^{*}}\right)}^{m\cdot p_{ACB,m}^{*}-1}}{L_{DS,m}^{*}}$$

$N_{L\_succ,m}$ is the number of successfully transmitted preambles in state m, which can be expressed as the product of the preamble utilization rate and the total number of preambles:

(27) $$N_{L\_succ,m}=m\cdot p_{ACB}^{*}{\left(1-\frac{1}{L_{DS,m}^{*}}\right)}^{m\cdot p_{ACB}^{*}-1}$$

Therefore, the average number of successfully transmitted preambles can be represented as:

(28) $$E\left[N_{L\_succ}\right]=\sum_{m=1}^{M}\pi_{m}\cdot m\cdot p_{ACB,m}^{*}{\left(1-\frac{1}{L_{DS,m}^{*}}\right)}^{m\cdot p_{ACB,m}^{*}-1}$$

It is not difficult for us to obtain the average number of preambles allocated to DS devices and the average value of ACB factor for DS devices within each slot. The expressions can be represented as:

(29) $$E\left[N_{L\_DS}\right]=\sum_{m=1}^{M}L_{DS,m}^{*}\cdot \pi_{m}$$

(30) $$E\left[p_{ACB}\right]=\sum_{m=1}^{M}p_{ACB,m}^{*}\cdot \pi_{m}$$

Combining Equation (28) with Equation (29), The average preamble utilization rate for DS devices in a slot can be obtained as:

(31) $$E\left[P_{L\_succ}\right]=\frac{E\left[N_{L\_succ}\right]}{E\left[N_{L\_DS}\right]}=\frac{\sum_{m=1}^{M}\pi_{m}\cdot m\cdot p_{ACB,m}^{*}{\left(1-\frac{1}{L_{DS,m}^{*}}\right)}^{m\cdot p_{ACB,m}^{*}-1}}{\sum_{m=1}^{M}L_{DS,m}^{*}\cdot \pi_{m}}$$

Combining Equation (4) with Equation (5), the probability of successfully access for a DS device in state m can be expressed as:

(32) $$P_{D\_succ,m}=p_{ACB,m}^{*}\cdot P_{access,m}=p_{ACB,m}^{*}\cdot {\left(1-\frac{1}{L_{DS,m}^{*}}\right)}^{m\cdot p_{ACB,m}^{*}-1}$$

therefore we can obtain the number of successful DS devices in state m as:

(33) $$N_{D\_succ,m}=m\cdot p_{ACB,m}^{*}\cdot {\left(1-\frac{1}{L_{DS,m}^{*}}\right)}^{m\cdot p_{ACB,m}^{*}-1}$$

then the expected number of successful DS devices in a slot is:

(34) $$E\left[N_{D\_succ}\right]=\sum_{m=1}^{M}\pi_{m}\cdot m\cdot p_{ACB,m}^{*}\cdot {\left(1-\frac{1}{L_{DS,m}^{*}}\right)}^{m\cdot p_{ACB,m}^{*}-1}$$

The expected number of active devices is:

(35) $$E\left[N_{D,m}\right]=\sum_{m=1}^{M}\pi_{m}\cdot m$$

Thus, we can obtain the average access successful rate for DS devices that attempts to access as:

(36) $$E\left[P_{D\_succ}\right]=\frac{E\left[N_{D\_succ}\right]}{E\left[N_{D}\right]}=\frac{\sum_{m=1}^{M}\pi_{m}\cdot m\cdot p_{ACB,m}^{*}\cdot {\left(1-\frac{1}{L_{DS,m}^{*}}\right)}^{m\cdot p_{ACB,m}^{*}-1}}{\sum_{m=1}^{M}\pi_{m}\cdot m}$$

from Equation (6) and Equation (35), the average delay of DS devices can be derived as:

(37) $$E\left[T_{delay}\right]=\frac{T_{slot}}{E\left[P_{D\_succ}\right]}=\frac{T_{slot}\cdot \sum_{m=1}^{M}\pi_{m}\cdot m}{\sum_{m=1}^{M}\pi_{m}\cdot m\cdot p_{ACB,m}^{*}\cdot {\left(1-\frac{1}{L_{DS,m}^{*}}\right)}^{m\cdot p_{ACB,m}^{*}-1}}$$

## 5. Performance Evaluation

### 5.1. Model Verification

**Figure 4:**
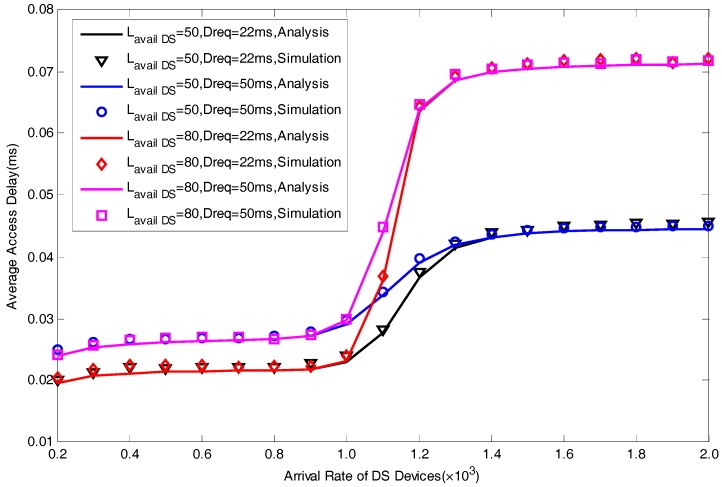
Average Access Delay with different $L_{avail\_DS}$ and $D_{req}$.

**Figure 5:**
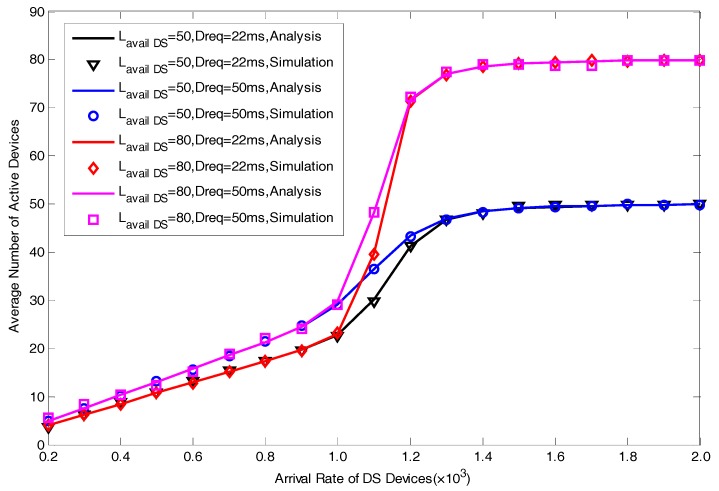
Average Number of Active Devices with different $L_{avail\_DS}$ and $D_{req}$.

**Figure 6:**
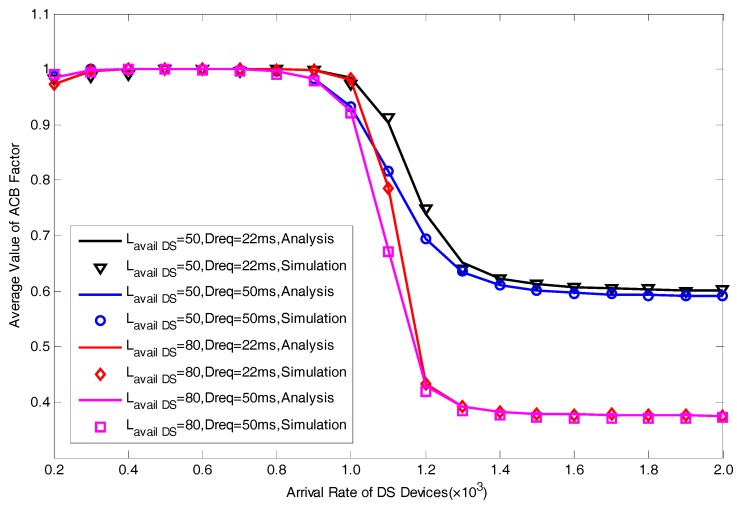
Average Value of ACB Factor with different $L_{avail\_DS}$ and $D_{req}$.

**Figure 7:**
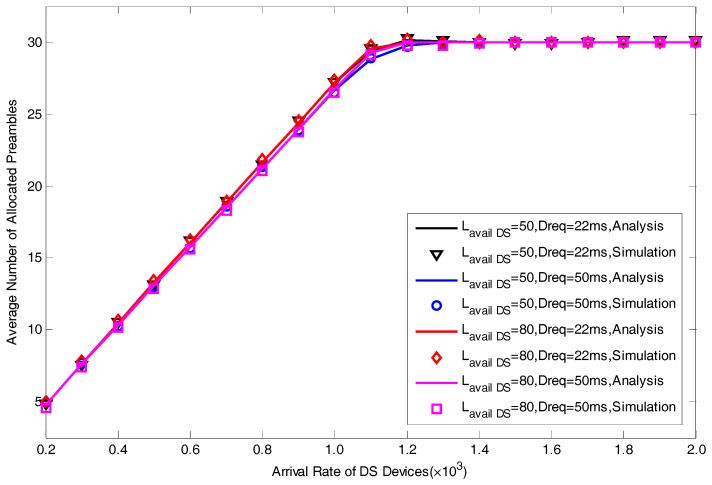
Average Number of Allocated Preambles with different $L_{avail\_DS}$ and $D_{req}$.

**Figure 8:**
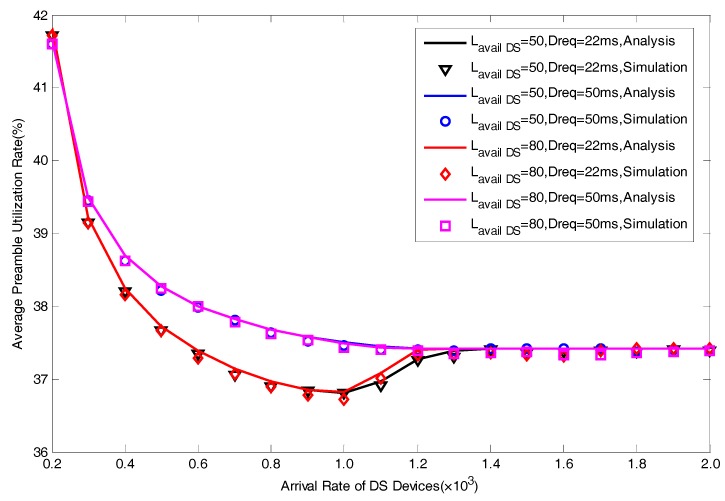
Average Preamble Utilization Rate with different $L_{avail\_DS}$ and $D_{req}$.

In this section, we present a series of simulation results to verify the correctness of the proposed analytical model in Figure 4, Figure 5, Figure 6, Figure 7 and Figure 8. The simulation results are obtained by MATLAB. We focus on five performance indexes, i.e., average number of allocated preambles, average value of $p_{ACB}$, average preamble utilization rate, average number of active devices and average access delay for DS devices as expressed by Equations (29)–(31), Equation (35) and Equation (37). It should be noted that in the actual scene, the value of M is far greater than $L_{avail\_DS}$ since we are considering the simultaneous DS devices’ access environment. However, in order to facilitate the model verification, we take a small value of M=30. In addition, to fully explain the results, the value of $L_{avail\_DS}$ is not set according to the actual configuration of LTE-A system. The specific parameters of model verification settings are listed in Table 1. 

From Figure 4, Figure 5, Figure 6, Figure 7 and Figure 8, we can find that the curves of our analysis model are basically consistent with the simulation curves. The curves can well exhibit the influence of different parameters on the performance of the system in our proposed scheme. Figure 4 shows the effect of different parameters in terms of average access delay. We set two value of delay requirements, i.e., $D_{req}=22\mathrm{ms}$ space between numbers and units and units not in italic and $D_{req}=50\mathrm{ms}$. It can be seen clearly that the smaller the value of $D_{req}$ is, the better the delay performance will be, this reflects the good performance in satisfying the delay requirement of our scheme. A larger value of M means a larger maximal number of active devices in each slot, thus it will lead to a greater peak delay.

Average number of active devices varying different values of $D_{req}$ and M is depicted in Figure 5. Through comparison of the results of M=50 with the results of M=80, we can find that if we set a larger number of M, the average number of active devices will increase. Furthermore, with the increase of $D_{req}$, the number of average active devices with a slot will become larger. 

Figure 6 and Figure 7 respectively show the performance of average value of $p_{ACB}$ and average number of allocated resources. In Figure 6, we can see that when we take a larger value of $D_{req}$, the average value of ACB factor will become smaller. With the increase of the arrival rate of DS devices, $p_{ACB}$ will decrease to a minimum value. As there will be more active devices when we take a larger value of M, the minimum value of $p_{ACB}$ for M=80 is much smaller than the value for M=50. The variation of average number of allocated preambles is shown in Figure 4. We can see that the curves for various $T_{req}$ and M are relatively close to each other. Whereas the value for $D_{req}=22\mathrm{ms}$ is slightly more than the value for $D_{req}=50\mathrm{ms}$, representing that there are more RA resources reserved for DS devices when we have a more stringent delay requirement. 

Average preamble utilization rate varying different values of $D_{req}$ and M is depicted in Figure 8. Through comparison of the result of $D_{req}=22\mathrm{ms}$ and the result of $D_{req}=50\mathrm{ms}$, If we set a larger value of $D_{req}$, the value of average preamble utilization rate will increase. The explanation for the results lies in the implementation process of the scheme that when the constraint of delay becomes looser, more preambles are allocated simply aiming at maximizing the preamble utilization rate with little attention to the access delay. Various value of M have no significant effects on the preamble utilization rate, it is because operation of the scheme won’t be changed by different M.

### 5.2. Performance Analysis

In this section, we report the validation results by comparing our proposed resource allocation and ACB scheme with the existing schemes for average access delay for DS devices, and average preamble utilization rate for DS devices and average number of preambles remained for DT devices. It should be noted that in order to simplify the demonstration of the performance of DT devices, we only considered the number of preambles remained for them, and the discussion of other performance indexes remains for our future work. As we have defined the total number of available preambles as $L_{total}$, the average number of preambles allocated to DS devices as $E\left[N_{L\_DS}\right]$ which has been described in Equation (29), we can obtain the average number of preambles remained for DT devices as:

(38) $$E\left[N_{L\_DT}\right]=L_{total}-E\left[N_{L\_DS}\right]$$

A typical configuration of the total number of available preambles in an RA slot is $L_{total}=64$ [25]. In order to guarantee the communication requirements of DS devices, we set $L_{avail\_DS}$ to a relatively large value 54. Considering that in actual system, the number of active devices within a slot can be much greater than the amount of available RA resources, we set M as 500. Other detailed parameters are set based on reference [25] and they are listed in Table 2.

With respect to the comparison targets, we consider the following conventional methods which are respectively expressed by scheme A, scheme B, scheme C and scheme D: A. fixed preamble allocation with fixed value of $p_{ACB}$ as described in [30]; B. fixed preamble allocation with dynamic tuning of $p_{ACB}$ as described in [33]; C. dynamic preamble allocation with fixed value of $p_{ACB}$ and D. dynamic preamble allocation with dynamic tuning of $p_{ACB}$ as described in [18]. In [30,33], the method for estimating the number of active devices is not discussed. In [18], load estimation is based on the preamble collision rate in previous slots. In order to facilitate performance comparison, we assume eNodeB has perfect knowledge of number of active devices for these schemes. In scheme A, we set $L_{DS}$ as its maximum value 54, $p_{ACB}$ as 0.2. In scheme B, the fixed number of preambles is set as 54, eNodeB will dynamically adjust the value of $p_{ACB}$ to $L_{avail\_DS}$/$N_{pa}$ to maximize the preamble utilization rate. In scheme C, $p_{ACB}$ is set as 0.2, eNodeB adjust the value of $L_{DS}$ using the similar mechanism as scheme B. In the implementation of scheme D, we take the value of parameter b in [18] as 1 and dynamically adjust $L_{DS}$ and $p_{ACB}$.

**Figure 9:**
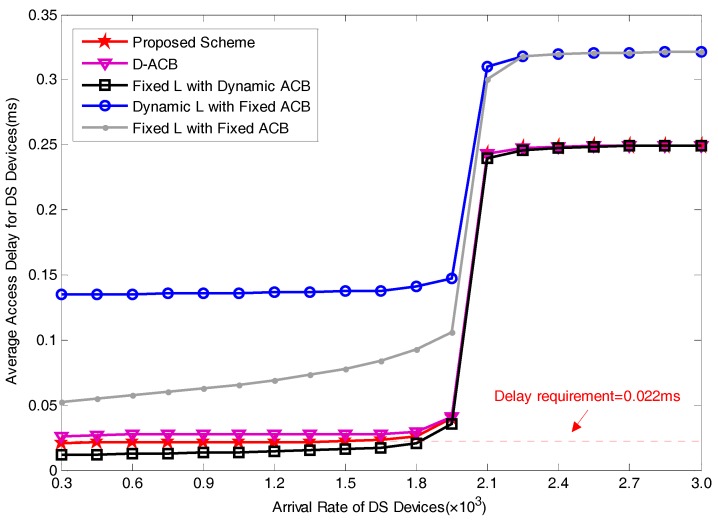
Average Access Delay for varying arrival rates.

**Figure 10:**
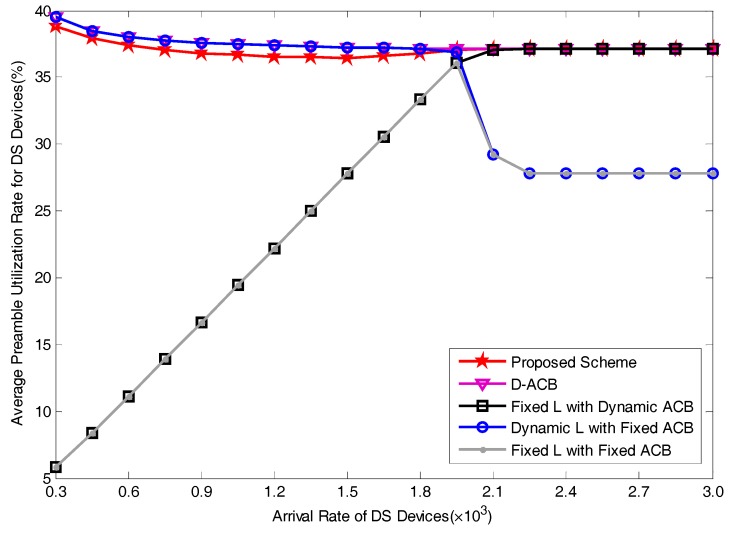
Average Preamble Utilization Rate for varying arrival rates.

Figure 9 and Figure 10 show the performances of average access delay and average preamble utilization rate for DS devices, respectively. In order to facilitate our comparison, we drew a straight line with the delay of 22ms in Figure 9. We can see from the result that among these five schemes, the scheme of fixed preamble allocation with dynamic $p_{ACB}$ shows better delay performances, it is because in this scheme, there are sufficient preamble resources reserved for DS devices. This scheme has the ability to satisfy the delay requirement in the wide range of the operating region. However, a significant disadvantage of fixed preamble allocation with dynamic $p_{ACB}$ scheme is that when the arrival rate of devices is relatively small, there will be a lot of idle preambles, i.e., preambles that are selected by no devices. As has been clearly shown in Figure 10, the redundancy of allocated preambles will obviously reduce the preamble utilization rate. With a larger value of the arrival rate, more devices will attempt to access the network, leading to an increase of average preamble utilization rate. When the number of available preamble resources are insufficient for active devices, the utilization rate remains at peak value.

Average access delays for DS devices obtained by scheme A and scheme C are relatively longer. In the operations of scheme A and scheme C, as $p_{ACB}$ is set to a fixed value 0.2, more devices will be blocked despite insufficient amount of RA resources. The improper access barring leads to an increase of average access delay. As the number of allocated preambles in scheme A is fixed as 54, active devices in scheme A have more preambles to consume than devices in scheme C. Therefore, average access delay of scheme A is shorter than that of scheme C. In these two schemes, when the number of active devices exceeds the maximum amount of available preamble resources, i.e., when the arrival rate of DS devices is larger than $1.8\times 10^{3}$, the fixed $p_{ACB}$ will lead to a longer access delay and a drop of preamble utilization rate due to the neglect of load variation. Compared with scheme A, the advantage of scheme C lies in it can dynamically adjust $L_{DS}$ based on load situation. Thus, it can maintain high preamble utilization rate when there are sufficient preamble resources. Similar as scheme B, the preamble utilization rate will gradually increase with the increase of arrival rate.

The performance curves of the D-ACB scheme is the most similar to that of our proposed scheme. Among pre-existing methods, D-ACB has the best delay performance. When comparing our proposed scheme with D-ACB scheme, the delay performance of our scheme is approximately 5 ms, i.e., 23%, better than D-ACB scheme. By comparing with the straight line of 22 ms, we can find that our scheme can effectively meet the delay requirement in a wide range of arrival rate. From the perspective of preamble utilization rate, our scheme is about one percentage point less than D-ACB scheme. Nevertheless, compared to the promotion of delay performance, the little drop of preamble utilization rate of our scheme is insignificant. It is worth noting that the comprehensive performances of our scheme and D-ACB scheme are better than most other schemes. 

**Figure 11:**
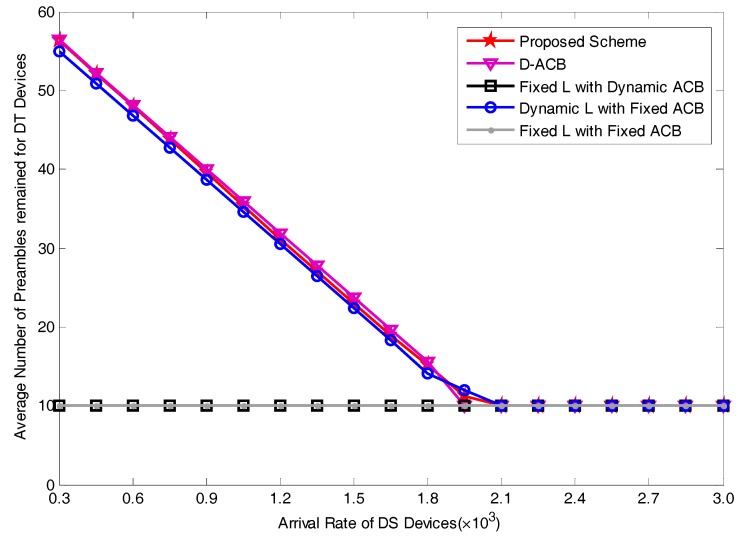
Average Number of Preambles for Pool 2 for varying arrival rates.

Figure 11 presents the number of preambles remained for DT devices, i.e., for Pool 2. We can observe that with the increase of the arrival rate of DS devices, average number of preambles remained for DT devices of scheme C, scheme D and our proposed scheme will gradually drop to a minimum value 10, as we have set the maximum number of available preambles for DS devices $L_{avail\_DS}$ to 54. The number of preambles remained for DT devices of scheme A and scheme B both maintain the value 10 for the reason that the number of preambles allocated to DS devices is set to a fixed value 54. From the curves of this figure, we can derive that our proposed scheme can save as much preambles as possible for DT devices for the reason that our scheme has considered the preamble utilization rate for DS devices. The analytical results present that our proposed scheme is better than scheme A and B in terms of the number of preambles remained for DT devices. When compared with D-ACB, our scheme exhibits almost the same performance with it for the reason that D-ACB has also paid attention to increase the preamble utilization rate. Our proposed scheme is slightly better than dynamic $L_{DS}$ with fixed $p_{ACB}$ scheme in terms of preambles remained for DT devices. However, compared with D-ACB and dynamic $L_{DS}$ with fixed $p_{ACB}$, our scheme shows its superiority in terms of average access delay as depicted in Figure 9, and this is the most important parameter for optimization of DS devices. In conclusion, our proposed scheme can not only satisfy the delay requirements of DS devices, but also save as much preambles as possible for DT devices. Above analysis shows that our scheme is best suited for the communication requirements of delay-sensitive devices. 

## 6. Conclusions

In this paper, we have proposed a novel random access scheme which is applicable to the scenario where delay-sensitive and delay-tolerant services coexist. Our novelty lies in the full consideration of the characteristics of delay-sensitive devices. For the high latency tolerance of delay-tolerant devices, we put this kind of equipment on a lower priority, and discuss the optimization problem from the perspective of delay-sensitive devices. By dynamically adjusting the ACB factor and the number of available preambles for delay-sensitive services, our proposed scheme can realize good performance in satisfying the QoS requirement as well as increasing the utilization rate of the random access resources allocated to them. High utilization rate ensures the resource efficiency therefore more resources can be leaved for delay-tolerant ones. Our proposed scheme can provide a promising idea for future research in the scene where devices with various QoS requirements coexists in M2M communications.

## Figures and Tables

**Table 1:** Parameter used in model verification.

Parameter	Value
M	30
$L_{avail\_DS}$	50, 80
$T_{slot}$	10 ms
$D_{req}$	22 ms, 50 ms
λ	200~2000 arrivals/s

**Table 2:** Parameters used in performance analysis.

Descriptions	Notation	Value
Maximum number of active devices to be handled in a slot	M	500
Arrival rate of DS devices	λ	300~3000 arrivals/s
Total number of available preambles in an RA slot	$L_{total}$	64
Maximum number of available preambles for DS Devices in an RA slot	$L_{avail\_DS}$	54
Length of a random access slot	$T_{slot}$	10 ms
Delay requirement of DS devices	$D_{req}$	22 ms
